# Crizotinib treatment for refractory pediatric acute myeloid leukemia with RAN-binding protein 2-anaplastic lymphoma kinase fusion gene

**DOI:** 10.1038/bcj.2016.52

**Published:** 2016-08-05

**Authors:** A Hayashi, R Tanoshima, S-I Tsujimoto, M Yanagimachi, M Takeuchi, K Sasaki, J Ikeda, R Kajiwara, S Ito, H Takahashi

**Affiliations:** 1 Department of Pediatrics, Yokohama City University, Yokohama, Japan; 2 Department of Pediatrics, Toho University School of Medicine, Tokyo, Japan

Crizotinib is an anaplastic lymphoma kinase (ALK) inhibitor, which has brought marked clinical benefits to patients with adult non-small cell lung cancer with *ALK* rearrangement.^1^ The *ALK* gene is located on chromosome 2p23 and its rearrangements with variable fusion partners have been identified in various malignant diseases such as anaplastic large cell lymphoma, inflammatory myofibroblastic tumors and neuroblastoma.^2^ The potential efficacy of crizotinib for these diseases has been reported.^2, 3, 4^ Myeloid neoplasms with *ALK* rearrangement are uncommon but several reports indicate that crizotinib is effective for these diseases.^5, 6^ The efficacy of an ALK inhibitor on leukemia cells with *ALK* rearrangement has been shown *in vitro*.^6^ Furthermore, crizotinib reduced leukemia cells in acute myeloid leukemia (AML) with *ALK* rearrangement.^5^

Here we report a pediatric patient with relapsed and refractory AML with RAN-binding protein 2 (*RANBP2*)-*ALK* fusion with monosomy 7, who achieved complete remission (CR) after treatment with crizotinib and allogeneic hematopoietic cell transplantation (allo-HCT).

A previously healthy 2-year-old female developed fatigue and a respiratory disorder. Her blood tests showed marked leukocytosis and anemia, and she was referred to our department. Her laboratory results were as follows: hemoglobin level 3.5 g/dl, platelet count 10 × 10^3^/μl and leukocyte count 159 × 10^3^/μl with 39% blast cells. A bone marrow aspirate showed hypercellularity with leukemia cells. The patient was diagnosed as acute myelomonocytic leukemia. The karyotype determined by G-banding was 45,XX,inv(2)(p23q13), which indicates *ALK* rearrangement, in all 20 cells analyzed. Other gene abnormalities, including monosomy 7, were not detected.

The patient was treated in accordance with Japanese Pediatric Leukemia/Lymphoma Study Group AML-05 protocol.^7^ After induction therapy, hematological CR was achieved and further G-banding analysis revealed a normal karyotype. The first relapse occurred 6 months after diagnosis, during consolidation therapy. In addition to inv(2)(p23q13), monosomy 7 was identified by fluorescence *in situ* hybridization. The patient received IDA-FLAG (Idarubicin, 10 mg/m^2^, on days 1 and 2; fludarabine, 30 mg/m^2^, on days 1–5; and cytarabine, 2 g/m^2^, once daily on days 1–5. Granulocyte colony-stimulating factor, 300 mg/m^2^, was administered daily beginning 1 day before the commencement of chemotherapy and continued until a neutrophil count of >500/μl), and azacitidine as salvage therapy; however, these proved ineffective.

Because the patient's leukemia cells carried *ALK* rearrangement and were refractory to conventional salvage therapy, we decided to administer crizotinib based upon its potential efficacy for the disease.^5^ After the approval of an institutional review board and written informed consent from her guardians, the patient received crizotinib, 280 mg/m^2^, twice a day, without concomitant chemotherapy except for intrathecal therapy (12 mg methotrexate, 25 mg cytarabine and 10 mg hydrocortisone). The dose of crizotinib used was determined based on phase I clinical trials by the Children's Oncology Group.^4^

Complete cytogenetic remission (disappearance of monosomy 7 in fluorescence *in situ* hybridization of bone marrow aspirate) was confirmed 51 days after the initiation of crizotinib. Severe adverse reactions did not occur except for nausea and vomiting. Subsequently the patient underwent allo-HCT from the 5/8 HLA-matched mother. Crizotinib was administrated for 55 days in total and discontinued 6 days before the initiation of a conditioning regimen, with total body irradiation of 12 Gy and 90 mg/m^2^ melphalan for 2 days. HCT was well tolerated, and neutrophil engraftment was achieved on day 29. Complete donor chimerism and the absence of monosomy 7 in a bone marrow aspirate was confirmed on day 28. CR has remained for more than 1 year after the HCT.

The presence of *RANBP2-ALK* fusion gene was confirmed by PCR with reverse transcription of a bone marrow sample at diagnosis and a relapse (Figure 1a).^8^ The *RANBP2-ALK* fusion gene disappeared after 51 days of administration of crizotinib and remained negative after HCT (Figure 1b).

This is the first pediatric case suggesting the effectiveness of crizotinib and HCT for relapsed and refractory AML with *ALK* rearrangement.

The potential efficacy of crizotinib for hematological malignancies with *ALK* rearrangement has been reported previously. In a pediatric phase I trial, seven out of eight patients with *ALK*-positive anaplastic large cell lymphoma achieved a CR after treatment with crizotinib.^4^ Maesako *et al.*^5^ reported the efficacy of crizotinib in reducing leukemia cells in AML with *ALK* rearrangement. However, resistance to crizotinib due to a secondary ALK kinase domain mutation occurred.^5, 9^ The authors consequently suggested the necessity of consolidation therapy using cytotoxic agents to achieve long-term remission.^5^ Allogeneic HCT is also a tolerable and curative option after crizotinib therapy, as reported in a case of refractory anaplastic large cell lymphoma.^3^ Our case achieved CR by crizotinib administration only and then successfully underwent subsequent HCT.

A *RANBP2-ALK* fusion gene combined with monosomy 7 may be responsible for a certain type of hematologic disorder, such as acute myelomonocytic leukemia, and be related to a poor prognosis.^10, 11^ Clinical data for our patient and five other previously reported patients^5, 10, 11^ showing a myeloid neoplasm with *ALK* rearrangement are summarized in Table 1. The neoplasms of all patients, including our case, were accompanied by monosomy 7 and were resistant to multidrug cytotoxic chemotherapy. Maxson *et al.*^6^ identified an oncogenic *ALK* point mutation in adult AML and pediatric B-cell acute lymphoblastic leukemia, and suggested that *ALK* mutations likely require other cooperating mutations for progression to leukemia. Monosomy 7, a potentially unfavorable prognostic factor in AML and juvenile myelomonocytic leukemia, may, in our patient, have a role as the cooperating mutation, together with *ALK* rearrangement, which leads to resistance to standard cytotoxic chemotherapy.

The prognosis of AML with monosomy 7 is dismal; however, our patient successfully achieved molecular CR by crizotinib monotherapy treatment and maintained CR for more than a year after HCT. Crizotinib can be viewed as a promising treatment option for those high-risk patients with *ALK* rearrangement. Therefore, we suggest examining *ALK* rearrangement and using crizotinib in patients with a refractory or relapsed myeloid neoplasm and chromosome 2p23 aberration. Further studies of the use of crizotinib in AML with *RANBP2-ALK* fusion gene are required to enhance our understanding of the contribution of crizotinib to the successful treatment of this disease.

## Figures and Tables

**Figure 1:**
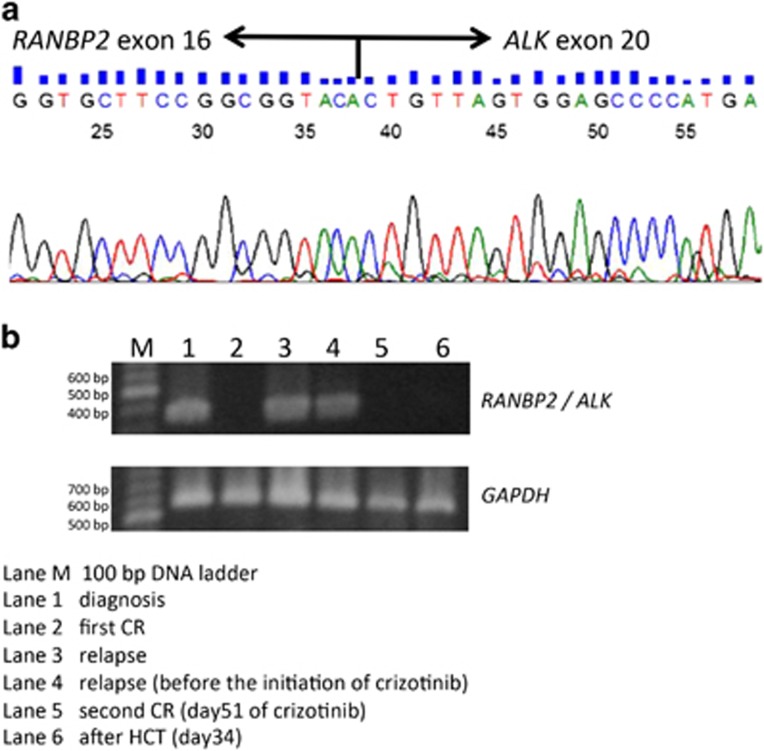
(**a**) Sanger sequencing of RANBP2-ALK fusion transcripts. The breakpoint lies between *RANBP2* exon 16 and *ALK* exon 20. (**b**) RT-PCR for *RANBP2-ALK* fusion transcripts in bone marrow aspirate samples. Total cellular RNA was extracted from leukemia blast cells with an RNeasy Mini Kit (QIAGEN, Tokyo, Japan). For RT-PCR analysis, 500 ng of total RNA was reverse-transcribed by PrimeScriptTM RT Master Mix according to the manufacturer's instructions (TaKaRa Bio, Tokyo, Japan). PCR reactions contained cDNA template, TaKaRa Ex Taq (TaKaRa Bio), 10 × Ex Taq buffer (TaKaRa Bio), dNTPs (TaKaRa Bio), forward primer (5′-CATTCTACACCGTCTCCTACCAG-3′) and reverse primer (5′-CGAGGTGCGGAGCTTGCTCAGC-3′) in a 50 μl reaction.^8^ The cycling conditions were as follows: one cycle of 94 °C for 5 min; 30 cycles of 94 °C for 30 s, 60 °C for 30 s and 72 °C for 30 s; and one cycle of 72 °C for 7 min. Sequencing of the PCR product was performed by FASMAC Co., Ltd. (Kanagawa, Japan). CR, complete remission; HCT, hepatopoietic cell transplantation; RT-PCR, reverse transcription-PCR.

**Table 1:** Characteristics of patients with myeloid malignancies and the *RANBP2-ALK* fusion gene

*Characteristic*	*This case*	*Maesako et al.*^5^	*Lim et al.*^10^	*Rottgers et al.*^11^ *Patient 3*	*Patient 4*	*Patient 6*
Diagnosis	AML (M4)	AML	AML (M4)	MDS or JMML	AML (M4)	JMML
Karyotype	46,XX,inv(2)(p23q13) [20] (at diagnosis) 45,XX,inv(2)(p23q13), -7 [5] (relapse)	46, XX, inv(2)(p23q13) [1]/, 45, idem, -7 [8]/,46, idem, -7, +mar [1]	45,XX,inv(2)(p23q13),-7 [20]	45,XY,inv(2) (p23q13),-7 [5]	46,XY,inv(2)(p23q13) [3]/, 45,idem,-7 [8]	45,XY,t(2;2) (p23;q11~13),-7
Age at diagnosis	2.8 Years	75 Years	31.4 Years	8 Years	16.3 Years	3.5 Years
Initial therapy regimen	VP-16, Ara-C, IDA	Ara-C, DNR, AZA (every 4 weeks)	Ara-C, DNR	Ara-C, DNR, VP-16	Ara-C, DNR, VP-16	Ara-C, DNR, VP-16
Response to initial therapy	Relapse 6 months after diagnosis, during consolidation therapy	Relapse after eight cycles of AZA	Induction failure	Recurrence of blasts on day 26	Early relapse after 6 months	Low persistent blast cells ranging between 2 and 6% during the next 4 months
Salvage therapy regimen	IDA+FLAG, AZA, Crizotinib	Crizotinib	Ara-C, MIT, VP-16	—	liposomal DNR, FLU, Ara-C	—
Response to crizotinib therapy	Molecular CR after 51 days crizotinib	Blasts disappeared from peripheral blood after 71 days crizotinib: blasts reappeared on day 135	—	—	—	—
HCT	HCT from HLA5/8 matched mother	—	MUD HCT	—	MFD HCT	MUD HCT
Clinical outcome	Alive 1 year after HCT	No data	Relapse 3 months after HCT; death 7 months after relapse	Early death due to infection	Alive 6 years after HCT	Alive 8 years after HCT

Abbreviations: AML, acute myeloid leukemia; Ara-C, cytarabine; AZA, azacitidine; DNR, daunorubicin; FLAG, fluradabine, cytarabine and granulocyte colony-stimulating factor; FLU, fludarabine; HCT, hematopoietic cell transplantation; JMML, juvenile myelomonocytic leukemia; MDS, myelodysplastic syndromes; MFD, matched family donor; MIT, mitoxantrone; MUD, matched unrelated donor; VP-16, etoposide.

## References

[bib1] Shaw AT, Engelman JA. ALK in lung cancer: past, present, and future. J Clin Oncol 2013; 31: 1105–1111. 23401436 10.1200/JCO.2012.44.5353 PMC4209068

[bib2] Hallberg B, Palmer RH. Mechanistic insight into ALK receptor tyrosine kinase in human cancer biology. Nat Rev Cancer 2013; 13: 685–700. 24060861 10.1038/nrc3580

[bib3] Cleary JM, Rodig S, Barr PM, Shinagare AB, Clark JW, Shapiro GI et al. Crizotinib as salvage and maintenance with allogeneic stem cell transplantation for refractory anaplastic large cell lymphoma. J Natl Compr Canc Netw 2014; 12: 323–326. 24616538 10.6004/jnccn.2014.0034

[bib4] Mosse YP, Lim MS, Voss SD, Wilner K, Ruffner K, Laliberte J et al. Safety and activity of crizotinib for paediatric patients with refractory solid tumours on anaplastic large-cell lymphoma: a Children's Oncology Group phase 1 consortium study. Lancet Oncol 2013; 14: 472–480. 23598171 10.1016/S1470-2045(13)70095-0 PMC3730818

[bib5] Maesako Y, Okumura A, Takeoka K, Kishimori C, Izumi K, Kamoda Y et al. Reduction of leukemia cell burden and restoration of normal hematopoiesis at 3 months of crizotinib treatment in RAN-binding protein (RANBP2)-anaplastic lymphoma kinase (ALK) acute myeloid leukemia. Leukemia 2014; 28: 1935–1937. 24850290 10.1038/leu.2014.166

[bib6] Maxson JE, Davare MA, Luty SB, Eide CA, Chang BH, Loriaux MM et al. Therapeutically targetable ALK mutation in leukemia. Cancer Res 2015; 75: 2146–2150. 26032424 10.1158/0008-5472.CAN-14-1576 PMC4453002

[bib7] Tomizawa D, Tawa A, Watanabe T, Saito AM, Kudo K, Taga T et al. Excess treatment reduction including anthracyclines results in higher incidence of relapse in core binding factor acute myeloid leukemia in children. Leukemia 2013; 27: 2413–2416. 23677335 10.1038/leu.2013.153

[bib8] Ma Z, Hill DA, Collins MH, Morris SW, Sumegi J, Zhou M et al. Fusion of ALK to the Ran-binding protein 2 (RANBP2) gene in inflammatory myofibroblastic tumor. Genes Chromosomes Cancer 2003; 37: 98–105. 12661011 10.1002/gcc.10177

[bib9] Takeoka K, Okumura A, Maesako Y, Akasaka T, Ohno H. Crizotinib resistance in acute myeloid leukemia with inv(2)(p23q13)/RAN binding protein 2 (RANBP2) anaplastic lymphoma kinase (ALK) fusion and monosomy 7. Cancer Genet 2015; 208: 85–90. 25766836 10.1016/j.cancergen.2015.01.003

[bib10] Lim JH, Jang S, Park CJ, Cho YU, Lee JH, Lee KH et al. RANBP2-ALK fusion combined with monosomy 7 in acute myelomonocytic leukemia. Cancer Genet 2014; 207: 40–45. 24613277 10.1016/j.cancergen.2013.12.003

[bib11] Rottgers S, Gombert M, Teigler-Schlegel A, Busch K, Gamerdinger U, Slany R et al. ALK fusion genes in children with atypical myeloproliferative leukemia. Leukemia 2010; 24: 1197–1200. 20428197 10.1038/leu.2010.18

